# *Mycobacterium tuberculosis*-Infected Hematopoietic Stem and Progenitor Cells Unable to Express Inducible Nitric Oxide Synthase Propagate Tuberculosis in Mice

**DOI:** 10.1093/infdis/jiy041

**Published:** 2018-02-17

**Authors:** Stephen T Reece, Alexis Vogelzang, Julia Tornack, Wolfgang Bauer, Ulrike Zedler, Sandra Schommer-Leitner, Georg Stingl, Fritz Melchers, Stefan H E Kaufmann

**Affiliations:** 1 Department of Immunology, Max Planck Institute for Infection Biology, Berlin, Germany; 2 Senior Group on Lymphocyte Development, Max Planck Institute for Infection Biology, Berlin, Germany; 3 Division of Immunology, Allergy and Infectious Diseases, Department of Dermatology, Medical University of Vienna, Austria

**Keywords:** inducible nitric oxide synthase, hematopoietic stem and progenitor cells, *Mycobacterium tuberculosis*

## Abstract

Persistence of *Mycobacterium tuberculosis* within human bone marrow stem cells has been identified as a potential bacterial niche during latent tuberculosis. Using a murine model of tuberculosis, we show here that bone marrow stem and progenitor cells containing *M. tuberculosis* propagated tuberculosis when transferred to naive mice, given that both transferred cells and recipient mice were unable to express inducible nitric oxide synthase, which mediates killing of intracellular bacteria via nitric oxide. Our findings suggest that bone marrow stem and progenitor cells containing *M. tuberculosis* propagate hallmarks of disease if nitric oxide-mediated killing of bacteria is defective.

## Teaser

We show that bone marrow stem and progenitor cells containing *Mycobacterium tuberculosis* induce hallmarks of tuberculosis if killing of intracellular bacteria is defective. Understanding the relative contribution of these cells in tuberculosis latency and reactivation could inform novel host-directed therapies.

Tuberculosis represents a devastating health problem with 10.4 million new cases and 1.7 million deaths in 2016 globally [1]. One compounding factor in efforts to control the global spread of tuberculosis is the ability of the causative bacterium, *Mycobacterium tuberculosis*, to cause latent tuberculosis infection in human hosts where bacteria persist in the absence of clinical signs of tuberculosis, but in the presence of an *M. tuberculosis*-specific immune response [2]. Individuals with latent tuberculosis infection remain at risk of developing active tuberculosis during their lifetime [3].

The circumstances of bacterial persistence during latent tuberculosis infection remain largely enigmatic. Recently, *M. tuberculosis* has been detected within mesenchymal [4] and hematopoietic [5] stem cell compartments in mice and humans. Both hematopoietic stem cells (HSC) and mesenchymal stem cells (MSC) are multipotent; HSCs giving rise to lymphoid and myeloid cell lineages in the blood [6] and MSCs giving rise to adipocytes, chrondrocytes, and osteoblasts, amongst other cell types [7]. HSCs or MSCs recovered from humans, with either latent tuberculosis infection or having been successfully treated for pulmonary tuberculosis, contain *M. tuberculosis* in a predominantly uncultivatable form [4, 5]. Evidence for the pathological context of carriage of *M. tuberculosis* within HSC or MSC compartments is currently lacking [8, 9]. In the present work, we used a murine model of tuberculosis where mice lack inducible nitric oxide synthase 2 (NOS2), an enzyme critical for defense against intracellular *M. tuberculosis*, with the aim of addressing the pathological context of this niche [8, 9].

## METHODS

### Bacterial Culture

*M. tuberculosis* strain H37Rv (American Type Culture Collection) was cultured until mid-log/late phase (OD_600nm_ 0.5–0.6) in Middlebrook 7H9 broth supplemented with ADC enrichment (BD Biosciences) and 0.05% Tween_80_ (vol/vol). Bacteria were harvested, resuspended in phosphate buffered saline (PBS) and stored at −80°C until use.

### Mice

All animal experiments were approved by the State Office of Health and Social Affairs Berlin (Landesamtes für Gesundheit und Soziales Berlin; approval number G0055/88). C57BL6 wildtype (wt) and C57BL6 *Nos2*^*−/−*^ mice (Charles River Laboratories) were bred in our facilities at the Max Planck Institute for Infection Biology, Berlin. For infection, 8-week old mice were anesthetized via intraperitoneal injection of ketamine (65 mg/kg), acepromazine (2 mg/kg), and xylazine (11 mg/kg) and 10^4^*M. tuberculosis* organisms were injected into the ear dermis.

### Harvest of Bone Marrow and FACS Sorting

Mice were sacrificed 4 weeks postinfection by cervical dislocation and femora and tibiae were removed from hind legs. Bone marrow cells were harvested by flushing femora and tibiae with PBS. Bone marrow cells were separated into lin^+^ and lin^−^ fractions using a lineage cell depletion kit for mice according to manufacturer’s instructions (Miltenyi Biotec). Lin^−^Sca1^+^ cells were purified from lin^−^ cells using an anti-Sca1 Microbead kit (fluorescein isothiocyanate) (Miltenyi Biotec). For subsequent identification of cell fractions, lin^−^ cells were stained with the following antibodies: c-Kit (2B8), Sca1 (D7), CD150 (TC15-12F12.2) (eBioscience). Stained cells were sorted to >98% purity by fluorescence activated cell sorting (FACS) using an Aria II flow cytometer (BD Biosciences).

### *IS6110* PCR

For polymerase chain reaction (PCR) analysis, lin^+^ and lin^−^, LSK CD150^+^ and LSK CD150^−^ cellular fractions were pelleted, resuspended in ultrapure water, and heat-treated at 80°C for 20 minutes. PCR was performed directly on heat-treated samples using the primers 5′-CGTGAGGGCATCGAGGTGGC-3′ and 5′-GCGTAGGCGTCGGTGACAAA-3′ to amplify a 245-base pair fragment located within the *IS6110* insertion element present in the H37Rv genome.

### Bone Marrow Transfers

Harvested whole bone marrow cells, purified lin^+^ or Lin^−^Sca1^+^ cell preparations were pelleted and resuspended in PBS at 10^7^/mL, and 100 µL containing 10^6^ cells of whole bone marrow cells, purified lin^+^ cell preparations, or either 5 × 10^5^ or 5 × 10^4^ Lin^−^Sca1^+^ cell preparation, was transferred into untreated C57BL6 wt or C57BL6 *Nos2*^*−/−*^ via the tail vein. Mice receiving cell preparations were monitored for signs of weight loss, sacrificed at 8 weeks post–transfer, and spleen, lung, and bone marrow were harvested. Organs were homogenized in PBS-Tween_80_ 0.05% (vol/vol), plated at suitable dilutions on 7H11 agar plates supplemented with Middlebrook OADC Enrichment (Difco), and incubated at 37°C. Colonies growing on agar plates were enumerated after ≥3 weeks.

### Histology

Mice were sacrificed 8 weeks after cell transfer and lung and spleen tissue were fixed in PBS containing 4% w/v paraformaldehyde overnight at room temperature. Sections of formalin-fixed, paraffin-embedded tissue, 2–3 μm thick, were deparaffinized and subjected to hematoxylin and eosin (H&E) staining.

## RESULTS

**Figure 1.**
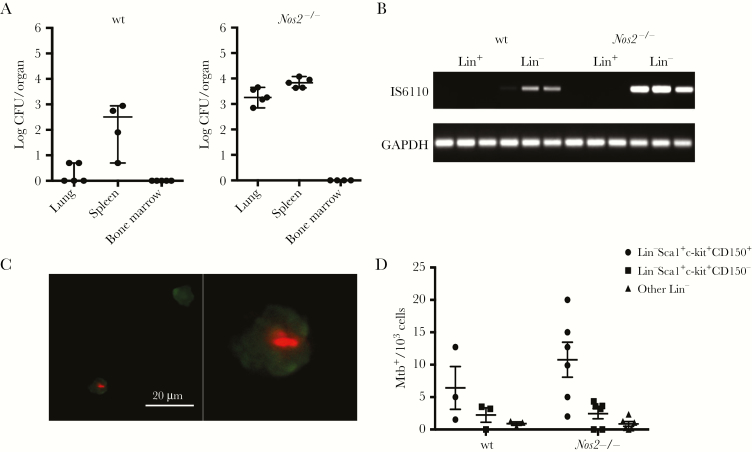
Wild-type (wt) and *Nos2*^*−/−*^ mice harbor uncultivable *Mycobacterium tuberculosis* (Mtb) in bone marrow stem and progenitor cell populations 28 days after dermal infection. *A*, Both wt and *Nos2*^*−/−*^ mice infected dermally with 10^4^ cultivatable *M. tuberculosis* showed a lack of cultivatable *M. tuberculosis* in the bone marrow at day 28 postinfection. Homogenates of lung and spleen from *Nos2*^*−/−*^ and wt mice revealed cultivatable *M. tuberculosis* at this time point (mean ± SEM; n = 5). *B,* lin^*−*^, but not lin^+^, cell preparations from harvested bone marrow are positive for the *M. tuberculosis*-specific *IS6110* DNA sequence by PCR (n = 3). All cellular preparations were positive for glyceraldehyde 3-phosphate dehydrogenase (GAPDH) showing comparable quantities of total DNA at day 28 postinfection. *C,* Representative staining of intracellular *M. tuberculosis* in hematopoietic stem and progenitor cells (HSPCs) by auramine-rhodamine stain. HSPC nuclei were stained using SYTOX green nucleic acid stain. Left panel shows one HSPC containing stainable *M. tuberculosis* and one uninfected HSPC. Right panel shows an enlarged image of the infected HSC (magnification × 1000). *D,* Frequency of stained intracellular *M. tuberculosis* in HSPC populations purified by FACS (mean ± SEM; n = 3 wt and n = 6 *Nos2*^*−/−*^). Highest frequency of intracellular *M. tuberculosis* was observed in Lin^*−*^Sca1^+^c-kit^+^CD150^+^ cell population, which was enriched for LT-pHSCs and ST-pHSCs. Abbreviation: CFU, colony forming unit.

We have previously described an experimental murine model of *M. tuberculosis* infection where *Nos2*^*−/−*^ mice are able to recapitulate hallmarks of human tuberculosis, including necrotizing granuloma pathology in the lung [10, 11]. As previously reported using this model, where an infectious dose of 10^4^ viable *M. tuberculosis* is injected into the dermis, we observed here systemic infection in *Nos2*^*−/−*^ mice with cultivatable *M. tuberculosis* in the spleen and lung at day 28 postinfection. Dermally infected wt mice showed cultivatable *M. tuberculosis* in the spleen at day 28 postinfection. We did not detect cultivatable *M. tuberculosis* from 5 × 10^7^ total bone marrow cells at day 28 postinfection from either wt or *Nos2*^*−/−*^ mice (Figure 1A).

To assay bone marrow cell populations from infected mice for presence of *M. tuberculosis* that could not be cultured, we sorted bone marrow cells into distinct compartments and probed DNA purified from these cells for the presence of *IS6110*, an insertion element present exclusively in *M. tuberculosis* complex strains, using PCR [12]. We separated cells based on expression of lineage markers to obtain a lin^+^ cell population and a negatively selected population lacking these markers (lin^*−*^), enriched for hematopoietic stem and progenitor cells (HSPCs). Using PCR, we identified *IS6110* by PCR in lin^*−*^ but not lin^+^ cell populations of the bone marrow (Figure 1B). To ascertain whether infections of lin^*−*^ cell populations were particular to dermal infection with *M. tuberculosis*, we examined the bone marrow of wt and *Nos2*^*−/−*^ mice 28 days after aerosol challenge with *M. tuberculosis*. In this setting, both wt and *Nos2*^*−/−*^ mice harbored approximately 10^6^*M. tuberculosis* in the lung and 1–100 culturable *M. tuberculosis* colony forming units (CFUs) in the bone marrow. We identified *M. tuberculosis* in both lin^*−*^ and lin^+^ populations using *IS6110* PCR, suggesting that presence of *M. tuberculosis* in lin^*−*^ cells was not particular to dermal infection with *M. tuberculosis*.

We then interrogated both wt and *Nos2*^*−/−*^ HSPC fractions for the presence of intracellular *M. tuberculosis* using rhodamin-auramin staining, which specifically detects intracellular *M. tuberculosis* using fluorescent microscopy (Figure 1C). We FACS-separated long-term (LT-HSC) and short-term repopulating hematopoietic stem cell progenitors (ST-HSC) as Lin^*−*^Sca1^+^c-kit^+^CD150^+^ cells, multipotent progenitors (MPP) as Lin^*−*^Sca1^+^c-kit^+^CD150^*−*^ cells, and remaining lin^*−*^ progenitors. We observed that numbers of *M. tuberculosis*-containing cells were highest (approximately 10 in 10^3^) in the fraction enriched for LT-HSC and ST-HSC populations (Lin^*−*^Sca1^+^c-kit^+^CD150^+^). The fraction enriched for MPP (Lin^*−*^Sca1^+^c-kit^+^CD150^*−*^) contained a markedly lower frequency of cells containing stainable acid-fast bacilli (>3 *M. tuberculosis*/10^3^ cells in MPP), while stainable *M. tuberculosis* were only rarely detectable in other lin^*−*^ cells (Figure 1D). We conclude that rhodamin-auramin–stained *M. tuberculosis* within HSPCs are detectable by PCR but do not form colonies on supplemented 7H11 agar.

**Figure 2.**
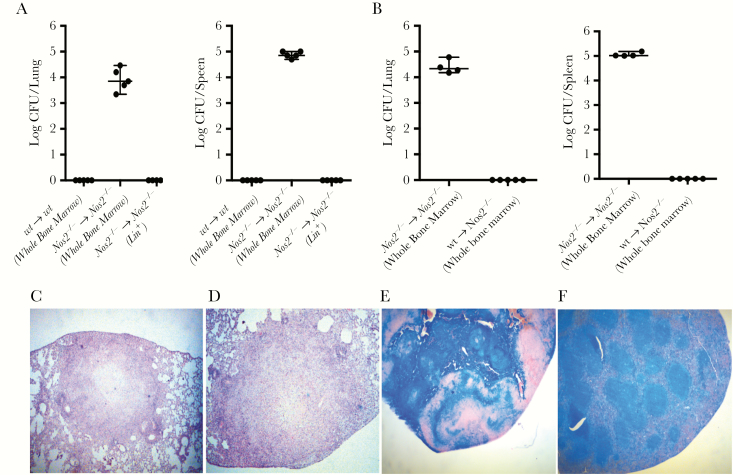
Transfer of bone marrow cells harboring uncultivatable *Mycobacterium tuberculosis* to naive mice results in tuberculosis. *A*, *Nos2*^*−/−*^ mice receiving 10^6^ whole bone marrow cells from dermal-infected *Nos2*^*−/−*^ mice at day 28 postinfection showed cultivatable *M. tuberculosis* in lung and spleen harvested at day 56 post-transfer, while wild-type (wt) mice receiving 10^6^ whole bone marrow cells from dermal-infected wt mice day 28 postinfection did not. Transfer of 10^6^ lin^+^ cells from dermal-infected *Nos2*^*−/−*^ mice day 28 postinfection to *Nos2*^*−/−*^ mice resulted in no cultivatable *M. tuberculosis* in lung and spleen at the equivalent time point (mean ± SEM; n = 5). *B, Nos2*^*−/−*^ mice receiving 10^6^ whole bone marrow cells from dermal-infected wt mice day 28 postinfection failed to show cultivatable *M. tuberculosis* in lung and spleen harvested at day 56 post-transfer. *C* and *D,* Hematoxylin and eosin staining of lung sections (magnification × 100) and (*E*) spleen sections (magnification × 50) from *Nos2*^*−/−*^ mice receiving 10^6^ whole bone marrow cells from dermal-infected *Nos2*^*−/−*^ mice day 28 postinfection reveal typical pathology associated with active tuberculosis. *F,* Spleen sections from *Nos2*^*−/−*^ mice receiving lin^+^ cells from dermal-infected *Nos2*^*−/−*^ mice show normal splenic architecture (magnification × 50). Abbreviation: CFU, colony forming unit.

We next investigated whether *M. tuberculosis* present in lin^*−*^ cellular fractions could propagate infection when delivered to naive recipient mice. We transferred 10^6^ bone marrow cells, containing approximately 1 × 10^4^ HSPCs harboring 5–10 *M. tuberculosis* stainable with rhodamin-auramin, from dermal-infected wt and *Nos2*^*−/−*^ mice to naive recipient mice via the tail vein of recipient mice and monitored recipients for signs of infection. Naive wt recipients of bone marrow cells from *M. tuberculosis* dermal-infected wt donors failed to show cultivable *M. tuberculosis* in spleen and lung at day 56 post-transfer. Remarkably, and in contrast to wt recipients receiving bone marrow cells from *M. tuberculosis* dermal-infected wt donors, *Nos2*^*−/−*^ recipients receiving bone marrow cells from *M. tuberculosis* dermal-infected *Nos2*^*−/−*^ donors harbored consistent numbers of cultivatable *M. tuberculosis* in lung, spleen, and liver (Figure 2A, Supplementary Figure 1*A*). Furthermore, we detected *IS6110* sequences from *M. tuberculosis* in lin^*−*^ fractions of bone marrow cells from these mice, as well as 1–100 *M. tuberculosis* CFUs (Supplementary Figure 1*B* and *C*). We conclude from these results that transfer of *M. tuberculosis*-infected bone marrow cells from *Nos2*^*−/−*^mice into *Nos2*^*−/−*^recipients by the intravenous route results in infection with cultivatable *M. tuberculosis* in lung, spleen, and bone marrow. When we transferred 10^6^*M. tuberculosis* -infected wt bone marrow cells from dermal-infected donors into *Nos2*^*−/−*^ recipients, we found no cultivatable *M. tuberculosis* in lung, spleen, liver, or bone marrow tissue harvested 8 weeks post-transfer (Figure 2B, Supplementary Figure 1*A* and *C*). These data indicate that the ability to propagate tuberculosis via *M. tuberculosis*-infected bone marrow cell transfer in mice was contingent on the inability of both the transferred bone marrow cells and of the host to express *Nos2*.

Finally, we evaluated the extent of pathology in *Nos2*^*−/−*^ recipients resulting from adoptive transfer of *Nos2*^*−/−*^ bone marrow cells from *M. tuberculosis*-infected donors. Organs were harvested 8 weeks after cell transfer and analyzed using hematoxylin and eosin staining. We observed demarcated granulomas (Figure 2C and D), similar to those observed after infection of *Nos2*^*−/−*^ mice via the dermal route with viable *M. tuberculosis* [11]. Spleens contained significant regions of cellular necrosis (Figure 2E) compared to uninfected controls (Figure 2F), indicating active tuberculosis.

Taken together, the bacterial load and pathology reveal that adoptive transfer of *Nos2*^*−/−*^ bone marrow cells, with *M. tuberculosis* present predominantly in HSPCs, can infect *Nos2*^*−/−*^ naive mice leading to the hallmarks of tuberculosis.

## Discussion

We show that *M. tuberculosis*-infected HSPCs in bone marrow are involved in propagating systemic hallmarks of the primary infection after adoptive cell transfer to naive mice, contingent on the inability of these cells to express NOS2. Intriguingly, although tuberculosis was propagated after adoptive transfer of 5 × 10^5^ HSPCs as microbead-purified Lin^*−*^Sac1^+^ cells (Supplementary Figure 3*A*–*C*), which contain a mixed population of LT-HSCs, ST-HSCs, and MPPs, transfer of 5 × 10^4^ infected LT-HSCs to *Nos2*^*−/−*^ mice did not result in tuberculosis (data not shown). We attribute this to the potential involvement of multiple bone marrow stem cell populations in triggering of tuberculosis from the bone marrow niche.

Although it has previously been shown that the vast majority of *M. tuberculosis* in HSC is in a noncultivatable state [5] and although the experiments presented here have not detected cultivatable *M. tuberculosis* in bone marrow cells from the *M. tuberculosis*-infected *Nos2*^*−/−*^ mice used as donors, we would not rule out that low numbers of cultivatable *M. tuberculosis* in HSPC were the origin of cultivatable *M. tuberculosis* in the *Nos2*^*−/−*^ recipients. Further characterization of the phenotypic status of *M. tuberculosis* in bone marrow stem cells, beyond that of cultivability on supplemented agar, could support our model as a system by which resuscitation of *M. tuberculosis* and transition to tuberculosis from latent tuberculosis infection can be studied further.

NOS2 is not expressed in resting cells [13] and thus NOS2 is not active in HSCs. NOS2 is induced by inflammatory cytokines in MSCs and controls growth of intracellular *M. tuberculosis* in vitro [14] and, similarly, could also be induced in more mature hematopoietic progenitors to kill intracellular *M. tuberculosis*, as they develop from HSC. Thus, wild-type bone marrow would retain *M. tuberculosis* only in the resting LT-HSC, as we have seen previously in *M. tuberculosis*-infected wild-type bone marrow cells [5]. However, bone marrow of *Nos2*^*−/−*^ mice might harbor *M. tuberculosis* in more-differentiated progenitors, such as MPP, as we have shown (Figure 1D). Moreover, tumor necrosis factor-alpha (TNF-α) augments NOS2 expression in MSCs in synergy with interferon-gamma (IFN-γ) in mice [15]. Because anti-TNF-α treatment is a known trigger of tuberculosis in humans, it is tempting to speculate that blocking of TNF-α may also abrogate the ability of later HSC progenitors and their lineage-positive cellular progeny, as well as MSCs, to control intracellular *M. tuberculosis*.

Our experiments support the essential role of NOS2 in control of *M. tuberculosis* in mice. This control might be exerted by the intracellular expression of NOS2 in hematopoietic cells, as well as through the interaction of the hematopoietic cells with NOS2-expressing endothelial and mesenchymal cells in hematopoietic niches. In contrast, the essential role of NOS2 for protection in human tuberculosis is more controversial and it is therefore tempting to speculate that additional antimycobacterial mechanisms could control *M. tuberculosis* reactivation in the early stages of progression from latent tuberculosis infection to active tuberculosis disease. Dissection and contextualization of the relative contribution of the individual cell types in this process could promote rational design of host-directed tuberculosis therapies in the future.

## Supplementary Data

Supplementary materials are available at *The Journal of Infectious Diseases* online. Consisting of data provided by the authors to benefit the reader, the posted materials are not copyedited and are the sole responsibility of the authors, so questions or comments should be addressed to the corresponding author.

Supplementary Figure 1

Supplementary Figure 2

Supplementary Figure 3

Supplementary Figure Legends

## Notes

***Financial support.*** This work was supported by European Commission FP7 project ADITEC (grant number HEALTH-F4-2011-280873 to S. H. E. K.), Bundesministerium für Bildung und Forschung inVAC (grant number 03ZZ0806A), and DFG Kosellek (grant number ME2764/1-1 to F. M.).

***Potential conflicts of interest.*** All authors: No reported conflicts of interest. All authors have submitted the ICMJE Form for Disclosure of Potential Conflicts of Interest. Conflicts that the editors consider relevant to the content of the manuscript have been disclosed.
