# Supplementary figures and images for: Mycobacterium tuberculosis-Infected Hematopoietic Stem and Progenitor Cells Unable to Express Inducible Nitric Oxide Synthase Propagate Tuberculosis in Mice
Source: J Infect Dis. 2018 Feb 17;217(10):1667–71. doi: 10.1093/infdis/jiy041 (PMC5913604; doi:10.1093/infdis/jiy041)

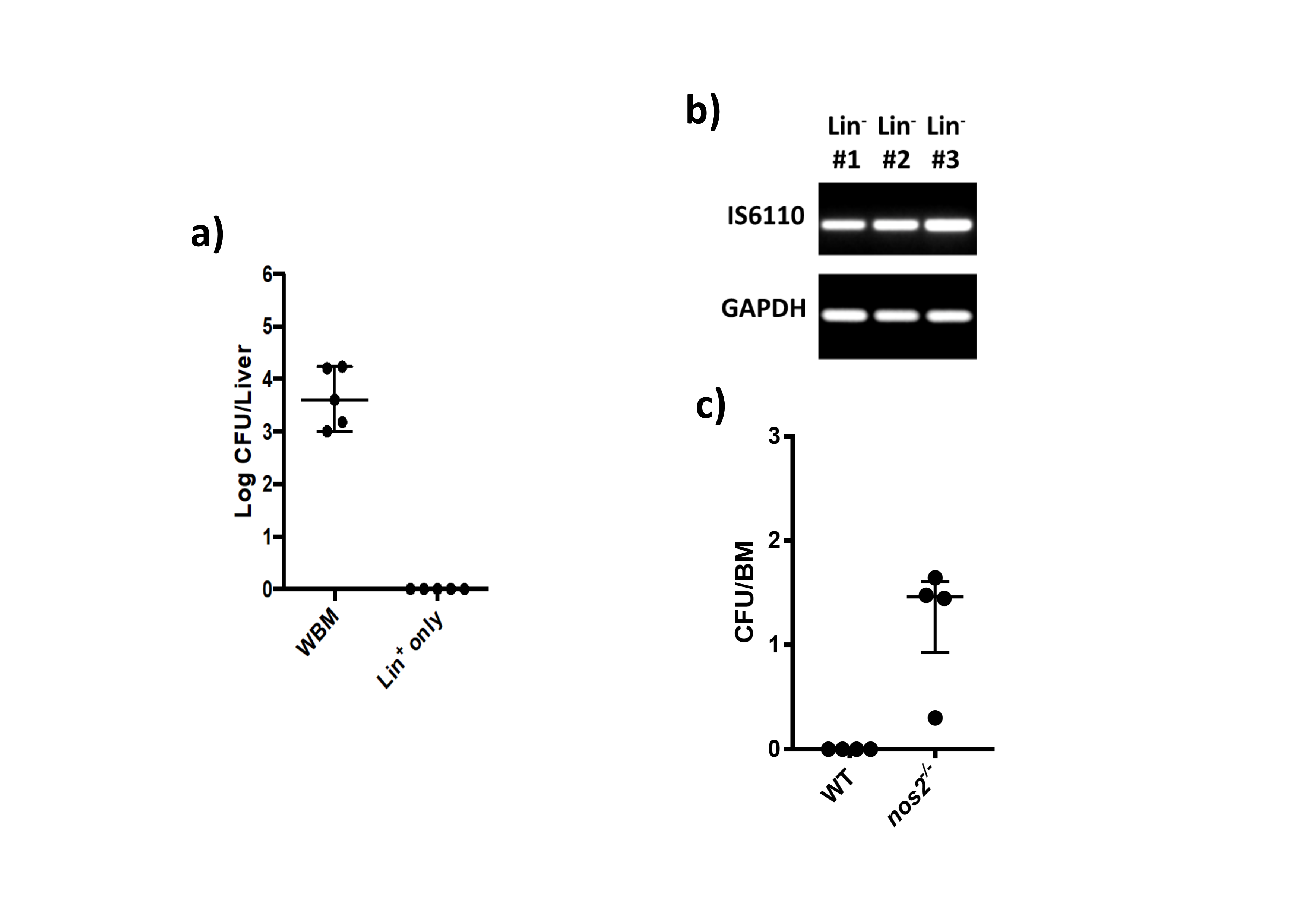

Supplement: Supplementary Figure 1 [file jiy041_suppl_supplementary_figure_1.png]

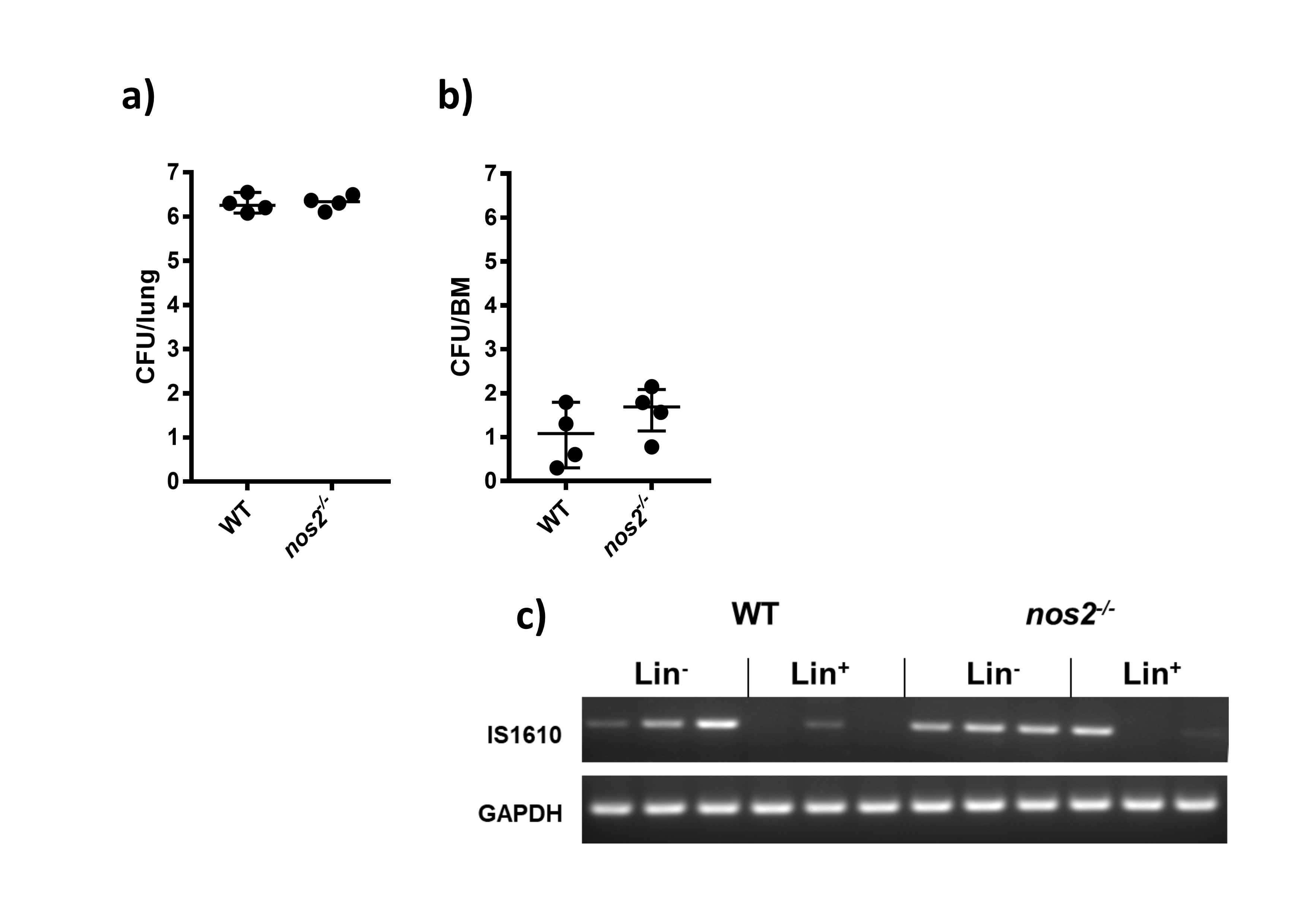

Supplement: Supplementary Figure 2 [file jiy041_suppl_supplementary_figure_2.png]

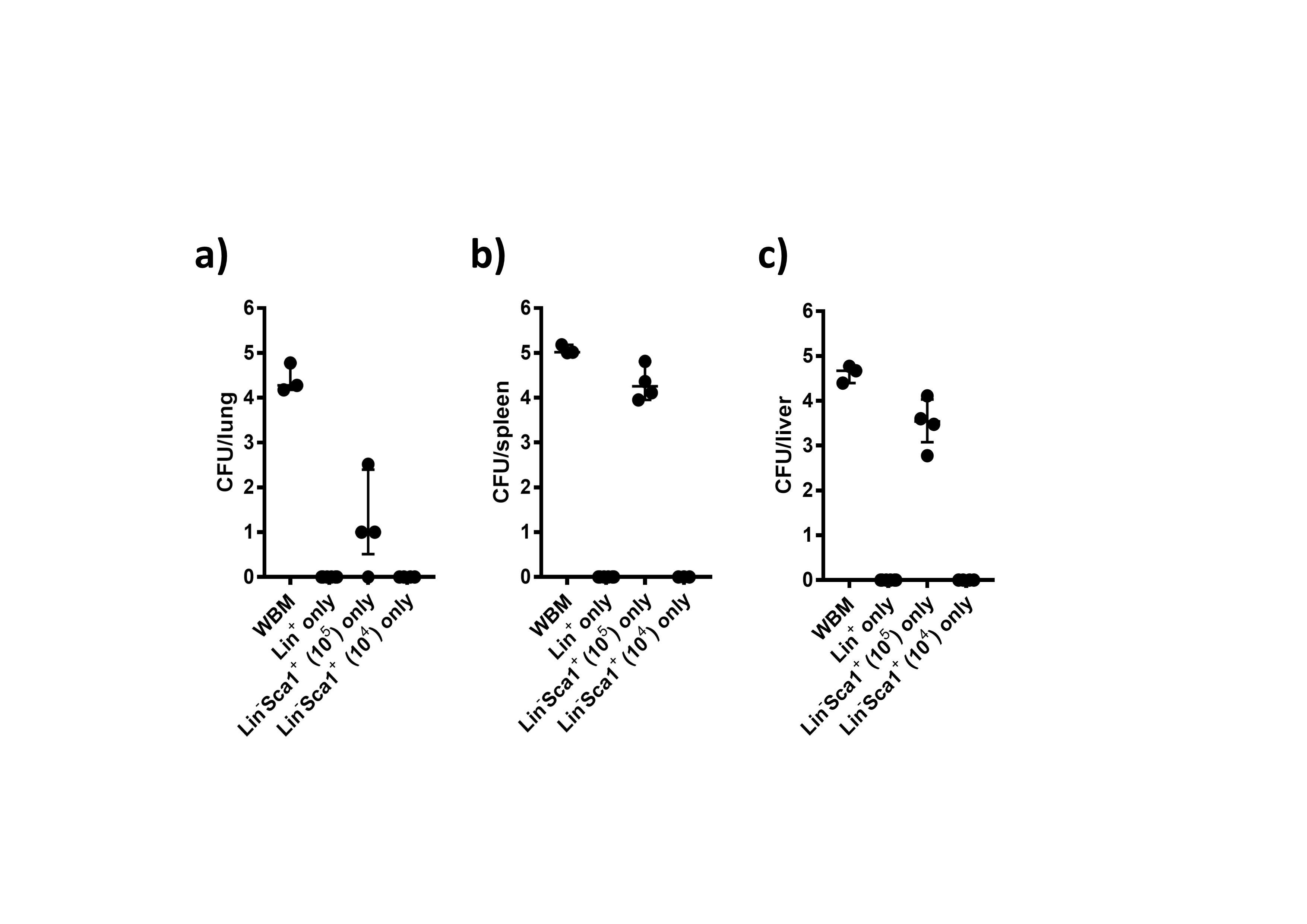

Supplement: Supplementary Figure 3 [file jiy041_suppl_supplementary_figure_3.png]
