# Supplementary material for: Mycobacterium tuberculosis-Infected Hematopoietic Stem and Progenitor Cells Unable to Express Inducible Nitric Oxide Synthase Propagate Tuberculosis in Mice
Source: J Infect Dis. 2018 Feb 17;217(10):1667–71. doi: 10.1093/infdis/jiy041 (PMC5913604; doi:10.1093/infdis/jiy041)
Supplement: Supplementary Figure Legends [file jiy041_suppl_supplementary_figure_legends.docx]

**Supplementary figure legend:**

**Supplementary Figure 1.** Transfer of bone marrow cells harboring uncultivatable Mtb to naïve mice results in TB. a) *Nos2^-/-^* mice receiving 10^6^ whole bone marrow cells (WBM) from dermal-infected *Nos2^-/-^* mice day 28 p.i. showed cultivatable Mtb in liver harvested at day 56 post-transfer. Transfer of 10^6^ lin^+^ cells resulted in no cultivatable Mtb in liver at the equivalent time point (mean ± SEM n=5). b) lin^-^ cell preparations from harvested bone marrow of *Nos2^-/-^* mice that received 10^6^ whole bone marrow cells from dermal-infected *Nos2^-/-^* mice day 28 p.i. are positive for the Mtb-specific *IS6110* DNA sequence by PCR. Glyceraldehyde 3-phosphate dehydrogenase (GAPDH) PCR shows comparable quantity of total DNA amounts (n=3). c) *Nos2^-/-^* mice receiving 10^6^ whole bone marrow cells (WBM) from dermal-infected *Nos2^-/-^* mice day 28 p.i. showed cultivatable Mtb in bone marrow (BM) harvested at day 56 post-transfer. WT mice receiving 10^6^ whole bone marrow cells (WBM) from dermal-infected WT mice day 28 p.i. showed cultivatable Mtb in bone marrow (BM) harvested at day 56 post-transfer (mean ± SEM n=4).

**Supplementary Figure 2.** Both WT and *Nos2^-/-^* mice infected via aerosol with ~20 cultivatable Mtb showed similar numbers of cultivatable Mtb in homogenates of lung (a) and in the bone marrow (b) at day 28 p.i. (mean ± SEM n=5). c) lin^-^ and lin^+^ cell preparations from bone marrow harvested from Mtb aerosol-infected WT and *Nos2^-/-^* at day 28 p.i. are positive for the Mtb-specific *IS6110* DNA sequence by PCR (n=3). Glyceraldehyde 3-phosphate dehydrogenase (GAPDH) PCR shows comparable quantity of total DNA amounts (n=3).

**Supplementary Figure 3.** HSPCs as microbead-purified Lin^-^Sac1^+^ cell preparations from bone marrow cells harvested from dermal-infected *Nos2^-/-^* mice day 28 p.i. propagate TB after transfer to naïve mice. *Nos2^-/-^* mice receiving 5 x 10^5^ Lin^-^Sac1^+^ bone marrow cells but not 5 x 10^4^ Lin^-^Sac1^+^ bone marrow cells showed presence of cultivatable Mtb in lung (a), spleen (b) and liver (c) harvested at day 56 post-transfer (mean ± SEM n=4).
